# Editorial: The Role of Eye Movements in Sports and Active Living

**DOI:** 10.3389/fspor.2020.603206

**Published:** 2020-10-22

**Authors:** Fabio Augusto Barbieri, Sérgio Tosi Rodrigues

**Affiliations:** ^1^Human Movement Research Laboratory (MOVI-LAB), Graduate Program in Movement Science, Department of Physical Education, School of Science, São Paulo State University (UNESP), Bauru, Brazil; ^2^Graduate Program in Movement Science, Laboratory of Information, Vision, and Action (LIVIA), Department of Physical Education, São Paulo State University (UNESP), Bauru, Brazil

**Keywords:** visual information, sports context, gaze behavior, movement control, eye-tracking

Eye movements are essential to collect accurate visual information from relevant scene locations, allowing optimal control of human movements in sports and active living. This Research Topic of the Frontiers in Sports and Active Living examines the role of the visual system in picking up the information necessary to guide action. It shows how essential gaze behavior is to timely collect accurate visual information from relevant scene locations and optimize control of human movements. Over the last four decades, substantial progress in research on eye movements and its application to sport and active living has been achieved through the use of more natural, ecologically valid research environments (in laboratory and *in situ* data collection situations), availability of newer technologies with higher measurement accuracy, increased experimental control, and novel approaches and analyses (Kowler, 2011; Discombe and Cotterill, 2015; Kredel et al., 2017; Moran et al., 2018).

Sport contexts are usually complex, often requiring fast actions, and eye movements are used to acquire adequate visual information. Particularly, gaze behavior of athletes reflects perception-based decision making and execution of motor responses involved in dynamic sports settings. Literature reveals strong evidence that skilled athletes show more efficient patterns of visual search than their less-skilled counterparts (e.g., Williams et al., 2005; Vickers, 2016). However, beyond describing relatively simple visual scanning paths during sports skills, many new theoretical questions relating to visual attention mechanisms, the relevance of visual information to action control and learning, gaze behavior training, as well as effects of new research methods have been investigated in the recent years. In short, researchers seem interested in answering not only *where* athletes and humans in general look, but *why* they do so when performing complex tasks (Kowler, 2011; Vater et al., 2020).

Articles in the present Research Topic highlight the growth of interest and the mentioned changes in this area. A rich combination of themes related to sport skills is presented by seven articles dedicated to perception and action characteristics of those skills. First, a perspective article by Klostermann et al. opens the debate on gaze behavior in sports. They analyzed the functionality of foveal and peripheral vision through three types of gaze behavior. The manuscript expands from the uniquely foveal tradition (“foveal spot”), which would optimize information acquisition from periphery (“gaze anchor” and “visual pivot”) in a complementary rather than a mutually exclusive manner.

Following on from this conceptual discussion, six articles investigated the following sport modalities: soccer, baseball, kendo, darts, and volleyball. Two studies focused on effects of environmental stimuli on gaze dynamics and motor performance. Paterson et al. analyze whether a task-irrelevant contextual information (i.e., moving advertisement behind the goal area) would distract players while performing a soccer penalty kick. They confirm that kickers' attention was disrupted during their goalkeeper-dependent strategy, highlighting systematic, direction-specific effects of advertisement motion on aiming task. Kishita, Uedo, Kashino et al. examine effects of varying speeds of a task-relevant stimulus (the ball) on visuomotor strategies used in baseball batting; authors stress that eye-head coordination depends on ball speed, but regardless of it, predictive saccade and quick head movement were temporally linked to bat-ball contact and hip motion. Next, two studies explore different gaze strategies. In another article, Kishita, Uedo, Kashino et al. focused on eye and head coordination patterns used by elite baseball players during batting in-game situations. They highlight that top batters delay initial saccades to obtain slightly more time acquiring visual information, as well as they may use head (instead of eyes) direction to encode bat-ball impact location. Kato explores how expert kendo fighters use a particular gaze strategy (referred above as “visual pivot”) during *in situ* sparring practice in offensive, defensive, and real match situations. He emphasizes that kendo experts keep gaze calmly stable on opponents' face but use peripheral vision strategy to obtain information from their body movements. Finally, two sport articles refer to distinct aspects involved in visual anticipation and gaze training. First, Lüders et al. examine effects of knowledge of an opponent's action preference on anticipation performance and gaze behavior during defense of volleyball attacks. They highlight influence of contextual information on anticipation, while systematic changes in the number of fixations and the duration of final fixation unobserved in defenders when they had preference information. Second, Neugebauer et al. examine characteristics of focus of attention during a quiet duration training for darts throwing. Even with similar throwing accuracy, authors show that visually instructed groups increased quiet eye duration whereas the kinesthetic group decreased it, suggesting perceptual and motor learning processes are dyssynchronous.

This Research Topic concludes with three articles bringing interesting applications of eye movements research to the context of public health and active living. Properly timed gaze behavior is critical to control the movements during active living; eye movements support adaptive motor control. First, a review article by Stuart et al. covers measurements of eye-movements in patients with mild traumatic brain injury, showing that there are no diagnostic criteria available for this injury. Next, Vargas et al. analyze body sway of sleep-deprived participants, highlighting that saccadic eye movements improve postural control even in sleep-deprived situation but are insufficient to avoid its deterioration due to sleep deprivation.

Finally, a brief report article by Baker et al. examine how individuals with Parkinson's disease perform walking turns with visual cues to promote anticipatory eye movements and, possibly, reduce the risk of falls. They emphasize that visual cue training is capable of changing from no anticipatory eye or segment movement to a pattern similar to that of neurotypical young adults, with craniocaudal rotation sequences during walking turns.

The multiplicity of contents presented in this Research Topic is illustrative of the growing interest in gaze behavior in sports and related activities and should stimulate new and innovative studies. As eye-tracking technologies and other motion measuring systems improve and become more popular in this field, many inspiring ideas and challenges are available for those interested in investigating the role of eye movements in sport and active living.

## Author Contributions

All authors listed have made a substantial, direct and intellectual contribution to the work, and approved it for publication.

## Conflict of Interest

The authors declare that the research was conducted in the absence of any commercial or financial relationships that could be construed as a potential conflict of interest.
